# Supplementary material for: Financial market regulation and corporate social responsibility: Evidence from China’s new asset management regulation
Source: PLoS One. 2025 May 15;20(5):e0323742. doi: 10.1371/journal.pone.0323742 (PMC12080806; doi:10.1371/journal.pone.0323742)
Supplement: Attachment — Submitted filename: Response to Reviewers.docx [file pone.0323742.s002.docx]

Response to reviewers

Dear editor and reviewers,

Thank you for offering us an opportunity to improve the quality of our submitted manuscript ***(Financial Market Regulation and Corporate Social Responsibility: Evidence from China's New Asset Management Regulation；Manuscript. Number.: PONE-D-24-48995)***. We appreciated the reviewers’ constructive and insightful comments very much. In this revision, we have addressed all these comments. We hope the revised manuscript has now met the publication standard of your journal.

We highlighted all the revisions in red color.

On the following pages, our point-to-point responses to the queries raised by the reviewers are listed.

**Suggestions from the editor：**

1. Please ensure that your manuscript meets PLOS ONE's style requirements, including those for file naming.

2. Please remove any funding-related text from the manuscript.

3. Please state what role the funders took in the study. If the funders had no role, please state: "The funders had no role in study design, data collection and analysis, decision to publish, or preparation of the manuscript."

Please include this amended Role of Funder statement in your cover letter; we will change the online submission form on your behalf.

4. At this time, please upload the minimal data set necessary to replicate your study's findings to a stable, public repository (such as figshare or Dryad) and provide us with the relevant URLs, DOIs, or accession numbers that may be used to access these data.

Response: Thank you for your suggestions. We have carefully addressed all the points as follows:

We have ensured that the manuscript meets PLOS ONE's style requirements, including appropriate file naming conventions.

All funding-related text has been removed from the manuscript as requested.

We have included the following amended statement regarding the role of funders in the study:

"The funders had no role in study design, data collection and analysis, decision to publish, or preparation of the manuscript."

This statement has also been added to the cover letter as instructed.

We have uploaded the minimal data set necessary to replicate our study's findings to [repository name, e.g., Figshare or Dryad] and provided the relevant [url: https://www.protocols.io/file/sj5yckdsp.xls].

We appreciate your guidance and hope that the revisions meet the journal's requirements. Please let us know if further adjustments are needed.

# Reviewer #1

**Comment 1:** The research gaps in the paper are not clearly defined. It would be helpful to elaborate on the specific areas where further research is needed. Providing a more detailed context will strengthen the paper's contribution to the field. There is a need for more citations to support the arguments presented. Specifically, consider integrating recent studies to demonstrate the relevance and novelty of your research.

**Response:** We sincerely appreciate your suggestion to emphasize the research gaps. Highlighting the unique contribution of our paper is indeed critical to its positioning within the academic literature. To address this, we have revised the introduction section to provide a more comprehensive research background related to financial market regulation and corporate social responsibility. Additionally, we have incorporated and cited recent, highly relevant literature to strengthen our contribution and clarify the unique aspects of our study. These references underscore the significance of our work in the context of evolving financial regulations and their impact on corporate practices. The newly added citations include:

1.	Dong Y, Dong M, Tan S, Ge R. Shadow banking, financial regulation, and bank risk in China. Financ Res Lett. 2024;63: 105293. doi:https://doi.org/10.1016/j.frl.2024.105293

2.	Jungo J, Madaleno M, Botelho A. The Effect of Financial Inclusion and Competitiveness on Financial Stability: Why Financial Regulation Matters in Developing Countries? Journal of Risk and Financial Management. 2022;15. doi:10.3390/jrfm15030122

3.	Ullah W, Zubir ASM, Ariff AM. Exploring the moderating effect of regulatory quality on the relationship between financial development and economic growth/economic volatility for developed and developing countries. Borsa Istanbul Review. 2024;24: 934–944. doi:https://doi.org/10.1016/j.bir.2024.04.015

4.	Delle Foglie A, Boukrami E, Vento G, Panetta IC. The regulators' dilemma and the global banking regulation: the case of the dual financial systems. Journal of Banking Regulation. 2023;24. doi:10.1057/s41261-022-00196-2

5.	Ofoeda I, Amoah L, Anarfo EB, Abor JY. Financial inclusion and economic growth: What roles do institutions and financial regulation play? International Journal of Finance and Economics. 2024;29. doi:10.1002/ijfe.2709

6.	Liu Q, Wu J. Strong financial regulation and corporate risk-taking: Evidence from a natural experiment in China. Financ Res Lett. 2023;54. doi:10.1016/j.frl.2023.103747

7.	Zhang B, Guo M. Strong financial regulation and the intelligent transformation of enterprises. Econ Anal Policy. 2025;85: 186–207. doi:https://doi.org/10.1016/j.eap.2024.11.017

8.	Jiang M, Zhou W, Song Y. Research on shadow banking, the new regulations on asset management and corporate finance. Stud of Int Financ. 2020;12: 63–72.

9.	Liu C. Disruption of corporate financialization and labor cost growth: Evidence from China's new asset management rules. PLOS One. 2023;18. doi:10.1371/journal.pone.0286683

**Comment 2:** Citation Style Errors. The citations are inconsistent with the required styles (APA 7 & IEEE). Below are the specific issues: APA 7th edition requires that author names be listed with the year in parentheses, for example, "Qin et al. (2023)".

Some citations are missing publication details or have incorrect formatting. For instance, "Yu et al. (2024)" and "Drucker, 2012" should be corrected.

Citation for Gao et al. (2023) is incomplete, and "Baron and Kenny's (1986)" should be fully cited with complete details.

Please ensure consistency in the citation style throughout the manuscript.

**Response:** Thank you for pointing out the inconsistencies in the citation style. We sincerely apologize for the oversight and have carefully revised all citations in the manuscript to ensure consistency and compliance with the required PLOS ONE citation format. We have also thoroughly reviewed the entire manuscript and have verified all references for accuracy and completeness.

**Comment 3:** Comments on the Use of the Difference-in-Differences (DID) Model:

Appropriateness of the DID Model:

The Difference-in-Differences (DID) model is an excellent choice when you are evaluating the causal impact of a policy intervention or treatment that affects a treatment group, while a control group is unaffected. It helps account for time trends that might affect both groups in the absence of the intervention, essentially comparing the differences in outcomes before and after the treatment for both groups.

Contextual Fit:

For the DID model to be appropriate, it is critical that the study satisfies the parallel trends assumption. This assumption posits that, in the absence of the treatment, the treatment and control groups would have followed similar trends over time. You should explicitly justify this assumption and, if possible, provide visual or statistical evidence (e.g., pre-treatment trends) to support that the treatment and control groups were on similar trajectories prior to the intervention.

Data Requirements:

The DID model requires data that covers pre-treatment and post-treatment periods for both the treatment and control groups. Please ensure that you have sufficient observations in both periods for both groups to draw reliable conclusions. Additionally, the model assumes that no other events or interventions during the study period are affecting the outcome, apart from the treatment being studied.

Possible Confounding Factors:

One potential issue with the DID model is the presence of confounding variables that might impact the treatment and control groups differently over time. It's crucial to control for variables that might influence the outcome, particularly if they vary across time or groups. You may want to include relevant covariates in your model or perform robustness checks to address any potential confounding.

Interpretation of Results:

The DID model provides an estimate of the average treatment effect for the treated units (ATT). Ensure that the interpretation of the coefficients is clear and that you're explaining the economic or policy implications of the results. It's also important to clarify whether your results reflect the short-term or long-term impact of the intervention.

Suggestions for Improvement:

If you are not already doing so, consider performing placebo tests or sensitivity analyses to ensure that the DID results are robust. You could also examine whether the parallel trends assumption holds by plotting the outcome variable for both groups before the treatment.

**Response:** Thank you for your insightful comments regarding the use of the Difference-in-Differences (DID) model in our study. We greatly appreciate your suggestions, as they have helped us enhance the rigor and clarity of our analysis.

We agree that satisfying the parallel trends assumption is critical for the validity of the DID model. To address this, we have conducted a parallel trends test and included statistical results to demonstrate that the treatment and control groups followed similar trajectories prior to the intervention. These results are presented in the revised manuscript.

As per your suggestion, we have performed a placebo test to ensure the robustness of our findings. The placebo test results confirm that there are no significant differences in the outcome variable during periods when the treatment should not have had any effect, further validating the causal interpretation of our results. These findings are also included in the revised text.

We appreciate your valuable feedback and trust that these revisions address your concerns effectively. Please let us know if there are any additional areas where further clarification or improvement is needed.

**Comment 4:** The manuscript is generally well-structured, but some sections lack clarity, especially in the Literature Review and Methodology sections. I suggest revising these sections for better flow and coherence. In particular, the explanation of the [specific model/approach] is somewhat confusing, and it would be helpful to simplify the description for readers unfamiliar with the technique.

Additionally, some figures and tables need clearer labels and better descriptions in the captions.

**Response:** Thank you for your valuable feedback regarding the structure and clarity of the manuscript. We appreciate your suggestions and have taken steps to improve the flow, coherence, and readability of the Literature Review and Methodology sections, as well as the presentation of figures and tables. We have revised the Literature Review section to enhance its logical flow and ensure that the key themes of relevant studies are clearly presented. To address the confusion regarding the explanation of the [specific model/approach], we have simplified the description, ensuring it is accessible to readers unfamiliar with the technique. We have reviewed all figures and tables in the manuscript and made some improvements.

**Comment 5:** Implications: The manuscript currently lacks a discussion on the implications of the findings. It is crucial to include the following:

Practical Implications: How do the findings apply to real-world scenarios? What recommendations can be made for practitioners or policymakers?

Theoretical Implications: How do the results contribute to advancing theory in the field? What new insights does the paper provide?

Social Implications: How does the research impact society? Are there any ethical, cultural, or social considerations arising from the findings?

**Response:** Thank you for your suggestion. The significance of the research is a crucial aspect of our paper. In the Introduction, we emphasize the theoretical importance of our study, while in the conclusion, we reassert both its theoretical and practical significance. Specifically, we highlight how our research contributes to the development of CSR theory and offers practical insights for firms and policymakers.

# Reviewer #2

**Comment 1:** Not enough information about the NAMR event. The authors fail to have a comprehensive introduction of the exogenous shock they employed. They should discuss the event in detail, including the precise time that the new policy was enacted, how the financial investment regulations are strengthened (in detail!), etc. More importantly, I am curious whether the policy was enacted nationwide concurrently or whether the authority started to have a trial in some provinces and extended it to other regions later. It would be vital to the DID setting in the empirical analysis.

**Response:** Thank you for your valuable suggestion. We recognize the importance of providing a comprehensive introduction to the NAMR event, and we have revised the manuscript to address this concern. We have added a dedicated section on the institutional background, which includes a detailed description of the NAMR event, including the exact timing of the policy enactment, the specific financial investment regulations that were strengthened, and the broader policy effects. To clarify, the policy was enacted nationwide at the same time, and there was no phased implementation or trial period in specific provinces before its nationwide rollout. We hope these additions clarify the policy context and strengthen the understanding of the empirical analysis. The revised section can be found in the end of introduction in the manuscript.

**Comment 2：**The selection of the treatment group is questionable. The authors use the level of financialization of non-financial firms before the event as a cutoff to separate the sample. It could be endogenous since firms with different levels of financialization are likely to have different preferences (motivation) for CSR. Thus, this setting may not capture the causal effect. I will suggest the authors look for cleaner treatment identification.

**Response:** We sincerely appreciate your advice for raising this important concern regarding the selection of the treatment group and the potential endogeneity issues associated with using the level of financialization of non-financial firms as the cutoff. Your insightful comment has allowed us to critically reassess and further strengthen our identification strategy.

We chose the level of financialization as the identifying variable for the following reasons:

Relevance to Policy Impact: The New Asset Management Regulation (NAMR) specifically targets the asset management activities of financial institutions, with a focus on curbing the "de-realization" of funds. This regulation has a direct impact on the financialization behaviors of firms, particularly those relying on financial assets or arbitrage. By using the pre-NAMR financialization level as the cutoff, we aim to capture the heterogeneous effects of the policy across firms with different financialization intensities, thereby providing a clearer identification of the NAMR's impact on firm behavior.

Alignment with Generalized DID Framework:

Second, the generalized DID model requires explicit treatment groups (firms significantly affected by the policy) and control groups (firms less affected by the policy). Firms with higher financialization levels are more likely to have relied on financial asset investments rather than production and business activities before the NAMR, making them more significantly affected by the regulation. Conversely, firms with lower financialization levels are less directly impacted. This distinction ensures that the treatment and control groups reflect the varying degrees of policy exposure.

Support from Prior Literature:

Several studies have successfully used financialization levels to define treatment and control groups in quasi-natural experimental settings, underscoring the feasibility of this approach. Representative studies include:

Xuan, S., Song, D., & You, G. (2024). Financial risk prevention and corporate green innovation: A quasi-natural experiment based on the new asset management regulation. Pacific-Basin Finance Journal, 88, 102566.

Li, M., & Huang, Y. (2024). Financial regulation and financial market stability: Evidence from stock price crash risk. Finance Research Letters, 69, 106196.

Jiang, T., & Wu, G. (2025). Can strict financial regulation improve analysts' forecast accuracy? Evidence based on a quasi-natural experiment in China. Finance Research Letters, 106752.

We acknowledge, however, the potential endogeneity concern raised by the reviewer. Firms with varying levels of financialization may indeed exhibit different preferences or motivations for corporate social responsibility (CSR), which could confound our analysis. To address this concern and further enhance the robustness of our findings, we have implemented the following adjustments:

Propensity Score Matching (PSM): We have redefined the treatment and control groups using a propensity score matching (PSM) approach. This method accounts for pre-existing differences in CSR preferences and ensures a more exogenous selection of the sample, minimizing bias.

Robustness Tests: To validate the causal inferences, we conducted additional robustness checks, including parallel trend tests, placebo tests, and variable substitution. The results consistently support our conclusions, demonstrating that our findings remain robust across different specifications.

**Comment 3：** The channels of the effect are not solid. Although the authors claim the reduction of financial investment profits, they do not illustrate the difference in financial profits of firms before and after the event. Additionally, the authors separate the hypothesis into two parts, the value of shareholders and stakeholders, where they assume the shareholders would advocate reducing the CSR expense after strengthening the regulation, while stakeholders would be the opposite. Do the results indicate that firms only care about the shareholders' interests while abandoning those for stakeholders? I will suggest the authors to more carefully diagnose their channels to explain this effect.

**Response:** We are grateful for the reviewer's thoughtful comments. To address the reviewer's concern about the lack of empirical evidence on the return on financial asset investments, we have conducted additional tests to examine the impact of the NAMR on the return on financial asset investments. The results, now presented in Table 8, show that the coefficient of Post × Prefin is significantly negative at the 10% level, indicating that the implementation of the NAMR significantly reduces firms' returns on financial asset investments. Moreover, the results in column (2) of Table 8 further demonstrate that both FinReturn and Post × Prefin are significantly negative, providing robust evidence that the NAMR reduces CSR investments by diminishing the returns on financial asset investments. These findings support our proposed channel that the return on financial asset investments under the NAMR directly influences their CSR spending decisions.

Regarding the division of our hypotheses into shareholder and stakeholder perspectives, we appreciate the reviewer's concern about the interpretation of our results. Our findings suggest that firms, under the constraints imposed by the NAMR, appear to prioritize shareholder interests by reducing CSR expenses. This behavior reflects firms' adaptive strategies in response to the regulatory shock and does not necessarily indicate a complete disregard for stakeholders. Rather, it highlights the tension between competing priorities in the immediate aftermath of regulatory changes.

We acknowledge that our study primarily captures the short-term effects of the NAMR, covering the four years following its implementation (2018–2022). During this period, firms may not have had sufficient time to adjust their CSR strategies to align with the new regulatory environment. This limitation has been incorporated into our discussion, and we have emphasized the need for future research to explore the long-term impacts of the NAMR. For instance, it would be valuable to examine whether firms' CSR activities eventually rebound as they adapt to the regulatory framework and reconcile the interests of shareholders and stakeholders.

**Comment 4：** The table format needs to be more professional. There is no caption to explain the regression econometrics of each table, and some inputs are not in the same line.

**Response:** Thank you for your helpful advice regarding the table format. We agree that presenting tables in a professional and clear manner is essential for enhancing the readability of the manuscript. In response to your comment, we have made the following revisions:

Table Captions: We have added captions to each table, providing clear explanations of the regression econometrics and the variables included.

Alignment and Consistency: We have corrected the alignment issues and ensured that all inputs are properly aligned in the same line, improving the overall presentation and consistency of the tables.

**Comment 5：**Paper writing needs to be polished.

**Response:** Thank you for your constructive advice. We understand the importance of clear and polished writing in academic papers. In response to your comment, we have carefully revised the manuscript to improve the clarity, fluency, and overall quality of the writing. We have focused on enhancing sentence structure, eliminating redundancy, and ensuring a smooth flow of ideas throughout the paper. Additionally, we have thoroughly checked for grammatical accuracy and coherence.

# Reviewer #3

**Comment 1:** It may be necessary to highlight the research significance of the paper in the abstract.

**Response:** Thank you for your valuable suggestion. We agree that the abstract serves as a crucial gateway for readers to understand the essence of the study. In response to your comment, we have revised the abstract to highlight the research significance more clearly. Specifically, we have emphasized the importance of our study at the end of the abstract, outlining its contributions to both theory and practice.

**Comment 2：**The Introduction exhibits a commendable structure and organization. However, it's pivotal to accentuate the novelty and unique contributions of this paper within the existing body of literature. This can be effectively showcased by delineating the primary contributions to the related works. Consider citing the most recent relevant literature to enrich the Introduction.

**Response:** We also appreciate your suggestion to highlight the novelty of our study compared to existing research. Undoubtedly, it's pivotal to accentuate the novelty and unique contributions of this paper within the existing body of literature. To address this, we have added a comparison with relevant literature in the introduction section, emphasizing our theoretical contribution. The references to the related studies are as follows:

1. Dong Y, Dong M, Tan S, Ge R. Shadow banking, financial regulation, and bank risk in China. Financ Res Lett. 2024;63: 105293. doi:https://doi.org/10.1016/j.frl.2024.105293

2.	Jungo J, Madaleno M, Botelho A. The Effect of Financial Inclusion and Competitiveness on Financial Stability: Why Financial Regulation Matters in Developing Countries? Journal of Risk and Financial Management. 2022;15. doi:10.3390/jrfm15030122

3.	Ullah W, Zubir ASM, Ariff AM. Exploring the moderating effect of regulatory quality on the relationship between financial development and economic growth/economic volatility for developed and developing countries. Borsa Istanbul Review. 2024;24: 934–944. doi:https://doi.org/10.1016/j.bir.2024.04.015

4.	Delle Foglie A, Boukrami E, Vento G, Panetta IC. The regulators' dilemma and the global banking regulation: the case of the dual financial systems. Journal of Banking Regulation. 2023;24. doi:10.1057/s41261-022-00196-2

5.	Ofoeda I, Amoah L, Anarfo EB, Abor JY. Financial inclusion and economic growth: What roles do institutions and financial regulation play? International Journal of Finance and Economics. 2024;29. doi:10.1002/ijfe.2709

6.	Liu Q, Wu J. Strong financial regulation and corporate risk-taking: Evidence from a natural experiment in China. Financ Res Lett. 2023;54. doi:10.1016/j.frl.2023.103747

7.	Zhang B, Guo M. Strong financial regulation and the intelligent transformation of enterprises. Econ Anal Policy. 2025;85: 186–207. doi:https://doi.org/10.1016/j.eap.2024.11.017

8.	Jiang M, Zhou W, Song Y. Research on shadow banking, the new regulations on asset management and corporate finance. Stud of Int Financ. 2020;12: 63–72.

9.	Liu C. Disruption of corporate financialization and labor cost growth: Evidence from China's new asset management rules. PLOS One. 2023;18. doi:10.1371/journal.pone.0286683

**Comment 3:** Please give more detailed descriptive stats.

**Response:** Thanks for your advice. We have revised the descriptive statistics section to provide more detailed information. We believe that these changes improve the overall quality of the manuscript and address your concern regarding the level of detail in the descriptive statistics.

**Comment 4:** Occasional grammar errors persist throughout the manuscript, notably concerning missing articles such as "the," "a," and "an." A thorough spellcheck to rectify these minor issues is advised. Additionally, some sentences tend to be overly long, impacting readability. Consider breaking these lengthy sentences into shorter, more digestible ones for enhanced readability.

**Response:** We sincerely apologize for any oversights in the initial submission and appreciate your guidance in identifying areas for enhancement. In response to your suggestions, we have conducted a comprehensive spellcheck to rectify missing articles and address minor grammatical issues. Additionally, we have restructured sentences to enhance the manuscript's readability.

To ensure the highest linguistic standards, we have also sought assistance from native English speakers for language corrections. we trust that these collaborative efforts have substantially improved the flow and language level of the manuscript.

# Reviewer #4

**Comment 1:** The article exhibits a conflation of concepts, equating Environmental, Social, and Governance (ESG) with corporate social responsibility (CSR), which occurs to varying degrees in both the theoretical and empirical analysis sections. In reality, while CSR and ESG are related, they are distinct. CSR refers to the social responsibilities that a company assumes in its business activities, whereas ESG encompasses the company's performance in environmental, social, and governance aspects.

**Response:** Thank you for your comment. In this study, we have chosen to use ESG as a proxy for measuring CSR. This decision is grounded in a well-established body of literature where ESG metrics are widely utilized as an empirical tool for evaluating CSR performance. ESG data, particularly Bloomberg's ESG scores, provide a comprehensive and consistent evaluation of companies' practices across environmental, social, and governance dimensions. These scores are based on robust quantitative and policy-relevant metrics, minimizing potential measurement errors in assessing CSR.

Several studies support the validity of using ESG metrics as a proxy for CSR. For instance, Gillan et al. (2010) utilized ESG scores to examine CSR practices, highlighting their relevance as a comprehensive measure of corporate social responsibility. Similarly, Wasiuzzaman et al. (2022) and Nollet et al. (2016) have demonstrated the efficacy of ESG metrics in empirical studies of CSR engagement.

Considering these precedents, our study employs ESG scores as an empirical measure of CSR to ensure a systematic and multidimensional evaluation of corporate responsibility. The following are proxies for CSR engagement through ESG scores：

1. Gillan, S., Hartzell, J. C., Koch, A., & Starks, L. T. (2010). Firms' environmental, social and governance (ESG) choices, performance and managerial motivation. Unpublished working paper, 10.
2. Nollet, J., Filis, G., & Mitrokostas, E. (2016). Corporate social responsibility and financial performance: a non-linear and disaggregated approach. economic Modelling, 52, 400-407.
3. Breuer, W., Müller, T., Rosenbach, D., & Salzmann, A. (2018). Corporate social responsibility, investor protection, and cost of equity: a cross-country comparison. Journal of Banking & Finance, 96, 34-55.
4. Egginton, J. F., & McBrayer, G. A. (2019). Does it pay to be forthcoming? Evidence from CSR disclosure and equity market liquidity. corporate social responsibility and environmental management , 26(2), 396-407.
5. Fahad, P., & Rahman, P. M. (2020). Impact of corporate governance on CSR disclosure. International Journal of Disclosure and Governance, 17(2), 155-167.
6. Wasiuzzaman, S., Uyar, A., Kuzey, C., & Karaman, A. S. (2022). Corporate social responsibility: is it a matter of slack financial resources or strategy or both? Managerial and Decision Economics, 43(6), 2444-2466.

**Comment 2:** In the appendix, the title of Figure 1 is incorrect.

**Response:** Thank you for pointing out the error in the title of Figure 1 in the appendix. We have carefully reviewed and corrected the title to ensure it accurately reflects the content of the figure. The revised version has been updated in the manuscript. We appreciate your attention to detail, which has helped improve the clarity and accuracy of our submission.

**Comment 3:** The specific results of the Propensity Score Matching (PSM) are not presented, making it impossible to assess the appropriateness of the PSM method used.

**Response:** Thank you for your suggestion. We understand the importance of providing detailed results to assess the appropriateness of the PSM method used. In response, we have included the specific results of the balance test for the PSM in the revised manuscript. These results are now presented in Table 6, which allows for a clearer evaluation of the matching process and ensures the robustness of our identification strategy.

Table 6. Balance test results

| Variables | Unmatched/  Matched | Mean | | %bias | %reduce\|bias\| | t-test | |
| --- | --- | --- | --- | --- | --- | --- | --- |
|  |  | Treated | Control |  |  | t | p>\|t\| |
| Size | U | -0.055 | 0.046 | -10.4 | 82.1 | -4.37 | 0.000 |
|  | M | -0.548 | -0.073 | 1.9 |  | 0.77 | 0.444 |
| Lev | U | -0.073 | 0.076 | -15.3 | 96.4 | -6.40 | 0.000 |
|  | M | -0.072 | -0.067 | -0.5 |  | -0.22 | 0.825 |
| ROA | U | 0.004 | 0.022 | -2.0 | 7.3 | -0.83 | 0.406 |
|  | M | 0.004 | -0.011 | 1.8 |  | 0.74 | 0.459 |
| Cashflow | U | -0.077 | 0.073 | -16.0 | 88.4 | -6.68 | 0.000 |
|  | M | -0.075 | -0.092 | 1.8 |  | 0.76 | 0.450 |
| Growth | U | -0.017 | -0.018 | -3.8 | 60.4 | -1.61 | 0.108 |
|  | M | -0.017 | -0.018 | 1.5 |  | 0.76 | 0.450 |
| Board | U | -0.090 | 0.089 | -18.2 | 98.0 | -7.64 | 0.000 |
|  | M | -0.090 | -0.086 | -0.4 |  | -0.15 | 0.881 |
| Indep | U | 0.041 | -0.055 | 10.2 | 86.0 | 4.26 | 0.000 |
|  | M | 0.041 | 0.054 | -1.4 |  | -0.57 | 0.570 |
| Top10 | U | -0.071 | 0.102 | -17.9 | 79.5 | -7.49 | 0.000 |
|  | M | -0.069 | -0.035 | -3.7 |  | -1.51 | 0.132 |
| Balance | U | 0.541 | -0.110 | 16.6 | 89.8 | 6.94 | 0.000 |
|  | M | 0.514 | -0.080 | -1.7 |  | -0.68 | 0.497 |
| INST | U | -0.086 | 0.110 | -20.0 | 97.9 | -8.39 | 0.000 |
|  | M | -0.084 | -0.080 | -0.4 |  | -0.17 | 0.867 |
| TMTPay | U | 0.025 | -0.099 | 12.9 | 91.5 | 5.42 | 0.000 |
|  | M | 0.024 | 0.013 | 1.1 |  | 0.45 | 0.655 |
| Separate | U | 4.735 | 6.318 | -19.3 | 97.2 | -8.06 | 0.000 |
|  | M | 4.740 | 4.694 | 0.5 |  | 0.24 | 0.809 |

**Comment 4:** Due to the author's conflation of ESG and CSR concepts, it is redundant to separately discuss the different dimensions of CSR, namely the E, S, and G aspects. It is recommended that these sections be removed.

**Response:** Thank you for your thoughtful advice., CSR is inherently a multidimensional concept, and each dimension may exhibit distinct responses to regulatory changes. By breaking CSR into its constituent parts, we are able to assess the specific impact of the NAMR on different aspects of CSR. In our study, we have decomposed CSR into a more structured and quantifiable framework, breaking down corporate responsibility into three specific dimensions: environmental performance (E), social performance (S), and governance performance (G). This categorization allows us to evaluate the differential effects of the NAMR on each of these dimensions and provides a clearer understanding of how the regulation influences various facets of corporate responsibility. For instance, a study published in Sustainability highlights the importance of analyzing environmental, social, and governance dimensions individually to understand their distinct relationships with corporate value.

https://www.mdpi.com/2071-1050/16/17/7376

**Comment 5:** The article overlooks the impact of other financial market policies in China when analyzing the effects of NAMR on corporate CSR, casting doubt on the reliability of the article's conclusions.

**Response:** We sincerely appreciate the reviewer's concern regarding the potential influence of other financial market policies in China on the effects of the NAMR, which could impact the reliability of our conclusions. In response, we have carefully considered the effects of two major factors, monetary policy, and U.S.-China trade frictions, and we have included additional analyses to validate their exclusion as confounding factors.

To address the potential impact of monetary policy, we used the broad money supply (M2) as a proxy. We incorporated an interaction term between M2 and Post in Model (1) to examine whether monetary policy could have influenced our results. The results, shown in column (1) of Table 5, indicate that monetary policy does not significantly affect the findings, supporting the robustness of our conclusions.

Similarly, to account for the effects of U.S.-China trade frictions, we included an interaction term between firms' export dependence (ED) and Post in Model (1). The results, presented in column (2) of Table 5, confirm that trade frictions between China and the U.S. do not significantly affect our findings.

These additional tests provide robust evidence that the observed changes in corporate CSR investments are primarily attributable to the NAMR rather than other concurrent policy influences. We have included this discussion in the revised manuscript to further strengthen the validity of our conclusions.

# Reviewer #5

**Comment 1:** Literature review could be more concise and focused on key arguments relevant to hypotheses.

**Response:** Thank you for your constructive advice. We have simplified and refined the literature review to make it more concise and focused. We have specifically emphasized key arguments that are directly relevant to the hypotheses of our study, ensuring that the review is more streamlined and aligned with the research objectives.

**Comment 2:** Discussion section requires stronger linkages between findings and broader theoretical implications.

**Response:** Thank you for your valuable advice. We have made significant revisions to address this concern, as outlined below:

In the revised Discussion section, we have explicitly linked our findings to broader theoretical frameworks, including shareholder primacy theory and stakeholder theory. Specifically, we discuss how the observed negative impact of stricter financial market regulation on CSR aligns with the shareholder value maximization view, where firms prioritize profitability over sustainability when faced with constrained financial resources. This highlights a broader theoretical tension between shareholder primacy and stakeholder theory, emphasizing the trade-offs firms face in balancing economic and social goals.

Additionally, we provide a nuanced analysis of how financial market regulation affects different dimensions of CSR (E, S, and G). Our findings reveal that the NAMR policy has a more pronounced impact on the E and G dimensions, with limited effects on the S dimension. We attribute this to the distinctive investment patterns of Chinese firms and the regulatory emphasis on environmental governance in recent years. This analysis offers insights into how regulatory interventions interact with corporate decision-making, contributing to the broader understanding of CSR dynamics in emerging markets.

**Comment 3：**Minor grammatical and stylistic issues need addressing to enhance overall clarity.

**Response:** Thank you for highlighting the grammatical and stylistic issues. We have thoroughly reviewed the manuscript and corrected all minor grammatical errors and stylistic inconsistencies. Specifically, we focused on improving sentence clarity, refining punctuation, and enhancing the overall flow of the text. We believe these revisions have significantly improved the clarity and quality of the manuscript.

**Comment 4：**More comprehensive discussion of study limitations and future research directions is warranted.

**Response:** Thank you for your valuable suggestion. We agree that a more comprehensive discussion of the study's limitations and future research directions is essential. In response, we have expanded this section to address the following points:

Geographic Context: We acknowledge that our study focuses on China, making the findings particularly relevant to developing countries. Given the differences in institutional and market dynamics between developed and developing economies, the applicability of our conclusions to developed countries may be limited. Future research could explore the impact of financial market regulation on corporate social responsibility (CSR) in developed economies, where market conditions and institutional frameworks differ significantly.

Timeframe of the Study: Due to data availability, the sample observation period in this study is relatively short, capturing only the immediate effects of the new asset management regulation. As a result, we are unable to comprehensively evaluate the medium- and long-term impacts of this policy on CSR. Future research could extend the observation period to assess whether the regulation has sustained effects on CSR and the broader real economy over time.

Contextual Factors: We also recognize that this study does not account for regional and industry-specific variations in financial market conditions, which may result in heterogeneous policy effects. Future research could incorporate contextual variables such as industry and regional characteristics to explore whether these factors moderate the policy's impact. Additionally, firm-specific resources and characteristics likely play a role in shaping CSR decisions, presenting another avenue for further investigation. We believe these revisions provide a more comprehensive perspective on the study's limitations and offer valuable directions for future research.

We tried our best to improve the manuscript, and we appreciate the editors/reviewers' warm work earnestly and hope that the correction will meet with approval. Once again, thank you very much for your comments and suggestions.

Thank you and best regards.

Yours sincerely,

LE ZHU

School of Accounting,

Shandong University of Finance and Economics,

Jinan 250000, Shandong, China.

E-mail: zhule19960717@gmail.com
