# Supplementary material for: Financial market regulation and corporate social responsibility: Evidence from China’s new asset management regulation
Source: PLoS One. 2025 May 15;20(5):e0323742. doi: 10.1371/journal.pone.0323742 (PMC12080806; doi:10.1371/journal.pone.0323742)
Supplement: Attachment — Submitted filename: Response_to_Reviewers_auresp_2.docx [file pone.0323742.s003.docx]

Response to reviewers

Dear editor and reviewers,

Thank you for offering us an opportunity to improve the quality of our submitted manuscript ***(Financial Market Regulation and Corporate Social Responsibility: Evidence from China's New Asset Management Regulation；Manuscript. Number.: PONE-D-24-48995R1)***. We appreciated very much the reviewers' constructive and insightful comments. In this revision, we have addressed all of these comments. We hope the revised manuscript has now met the publication standard of your journal.

We highlighted all the revisions in red color.

On the following pages, our point-to-point responses to the queries raised by the reviewers are listed.

# Suggestions from the editor：

1.Dear authors, Please address the comments of the reviewers in detail. In addition, please pay attention to the concepts of ESG and CSR and try to address these concepts in detail.

Response: Thank you for your valuable reminder. Following the reviewers' suggestions, we have provided a detailed explanation of the correlation between ESG and CSR in the variable description section. Additionally, we have clarified the differences between ESG and CSR, and we have elaborated on the scientific and rational basis for using ESG as a measure for CSR.

2.Please review your reference list to ensure that it is complete and correct. If you have cited papers that have been retracted, please include the rationale for doing so in the manuscript text, or remove these references and replace them with relevant current references. Any changes to the reference list should be mentioned in the rebuttal letter that accompanies your revised manuscript. If you need to cite a retracted article, indicate the article’s retracted status in the References list and also include a citation and full reference for the retraction notice.
Response: Thank you for your reminder regarding the reference list. We have reviewed and updated the references in response to the first-round reviewer's suggestions, which recommended enriching the research background and emphasizing the contributions of our study. The newly added citations include:

1. Dong Y, Dong M, Tan S, Ge R. Shadow banking, financial regulation, and bank risk in China. Financ Res Lett. 2024;63: 105293. doi:https://doi.org/10.1016/j.frl.2024.105293

2. Jungo J, Madaleno M, Botelho A. The Effect of Financial Inclusion and Competitiveness on Financial Stability: Why Financial Regulation Matters in Developing Countries? Journal of Risk and Financial Management. 2022;15. doi:10.3390/jrfm15030122

3. Ullah W, Zubir ASM, Ariff AM. Exploring the moderating effect of regulatory quality on the relationship between financial development and economic growth/economic volatility for developed and developing countries. Borsa Istanbul Review. 2024;24: 934–944. doi:https://doi.org/10.1016/j.bir.2024.04.015

4. Delle Foglie A, Boukrami E, Vento G, Panetta IC. The regulators’ dilemma and the global banking regulation: the case of the dual financial systems. Journal of Banking Regulation. 2023;24. doi:10.1057/s41261-022-00196-2

5. Ofoeda I, Amoah L, Anarfo EB, Abor JY. Financial inclusion and economic growth: What roles do institutions and financial regulation play? International Journal of Finance and Economics. 2024;29. doi:10.1002/ijfe.2709

6. Liu Q, Wu J. Strong financial regulation and corporate risk-taking: Evidence from a natural experiment in China. Financ Res Lett. 2023;54. doi:10.1016/j.frl.2023.103747

7. Zhang B, Guo M. Strong financial regulation and the intelligent transformation of enterprises. Econ Anal Policy. 2025;85: 186–207. doi:https://doi.org/10.1016/j.eap.2024.11.017

8. Jiang M, Zhou W, Song Y. Research on shadow banking, the new regulations on asset management and corporate finance. Stud of Int Financ. 2020;12: 63–72.

9. Liu C. Disruption of corporate financialization and labor cost growth: Evidence from China’s new asset management rules. PLoS One. 2023;18. doi:10.1371/journal.pone.0286683

In this revision, we have added new references to better explain the concepts of ESG and CSR and their interrelationship. These additional citations are as follows:

58 Carroll AB. The pyramid of corporate social responsibility: Toward the moral management of organizational stakeholders. Bus Horiz. 1991;34. doi:10.1016/0007-6813(91)90005-G

59. Yoon B, Lee JH, Byun R. Does ESG performance enhance firm value? Evidence from Korea. Sustainability (Switzerland). 2018;10. doi:10.3390/su10103635

60. Park JG, Park K, Noh H, Kim YG. Characterization of CSR, ESG, and Corporate Citizenship through a Text Mining-Based Review of Literature. Sustainability (Switzerland). 2023;15. doi:10.3390/su15053892

# Reviewer #2

**Comment 1**. There are still no captions for each table and figure.

Response：Thank you for your insightful comments. We have now added descriptive captions to every table and figure in the revised manuscript. the table now includes a note that summarizes its contents and explains the variables, and the figure has an accompanying caption that clearly outlines its data and significance.
**Comment 2:**The paper writing should be further polished. There are frequent typos and grammar errors in the paper. Additionally, the introduction and conclusion can be more concise while emphasizing the contribution of this paper to the existing literature.

Response：Thank you for your valuable advice. We have thoroughly proofread the manuscript to correct typos and grammatical errors, ensuring a polished and professional presentation throughout. We have revised both the Introduction and Conclusion to be more concise. We believe these revisions enhance the clarity, flow, and overall impact of the manuscript. Thank you again for your constructive suggestions.

# Reviewer #4

**Comment 1:**.ESG and CSR concepts are highly correlated. So, the author should explain the correlation between the two concepts in the variable description. And explain the differences between ESG and CSR.

Response：Thank you for your valuable advice. We fully agree with your suggestion to clarify the correlation between ESG and CSR, as well as to explain the differences between the two concepts. In the revised manuscript, we have provided a more detailed distinction between the two concepts, as follows:

"The key dependent variable in this study is corporate social responsibility (CSR). CSR encompasses corporations' voluntary efforts to exceed legal and financial obligations, contributing to stakeholders through ethical practices, social initiatives, and sustainability efforts [58]. Yoon et al. describe CSR as a broad, discretionary concept that includes social, ethical, and environmental activities beyond regulatory requirements [59]. However, measuring CSR has been a persistent challenge due to its subjective and qualitative nature. ESG emerged as a response to this challenge by standardizing and quantifying corporate responsibility efforts, making them comparable and transparent across firms and industries. As a result, ESG is not separate from CSR but rather an institutionalized mechanism that translates CSR commitments into measurable environmental, social, and governance indicators. Park et al. conduct a text-mining analysis and found that CSR and ESG are frequently discussed together, but their conceptual focus differs: CSR is qualitative and voluntary, focusing on corporate goodwill and ethical obligations. ESG is quantifiable and investment-driven, providing a framework that integrates corporate responsibility into financial and risk assessments [60]. In other words, ESG serves as a mechanism to operationalize CSR, ensuring that corporate responsibility efforts are systematically assessed and financially material to investors.

Given the strong correlation between CSR and ESG, ESG may serve as a proxy for CSR, offering more systematic coverage and improved data availability. We utilize Bloomberg's ESG composite score. The Bloomberg ESG measure minimizes measurement mistakes by offering a consistent and thorough evaluation of CSR behaviors. The ESG score assesses the quality and comprehensiveness of a firm’s environmental and social governance disclosure and reporting activities and is based on several quantitative and policy-relevant indicators. Compared to other ESG ratings, the Bloomberg ESG composite score is more comprehensive and includes sub-scores for the E, S, and G dimensions. Therefore, the higher the ESG score, the greater a firm’s engagement in CSR activities. Furthermore, the extensive usage of Bloomberg CSR rating scores in the body of current CSR research lends credence to the data's legitimacy and authenticity [61–64]."

We have added the following references to the revised manuscript:

58 Carroll AB. The pyramid of corporate social responsibility: Toward the moral management of organizational stakeholders. Bus Horiz. 1991;34. doi:10.1016/0007-6813(91)90005-G

59. Yoon B, Lee JH, Byun R. Does ESG performance enhance firm value? Evidence from Korea. Sustainability (Switzerland). 2018;10. doi:10.3390/su10103635

60. Park JG, Park K, Noh H, Kim YG. Characterization of CSR, ESG, and Corporate Citizenship through a Text Mining-Based Review of Literature. Sustainability (Switzerland). 2023;15. doi:10.3390/su15053892

Once again, thank you for your insightful comments. We look forward to your further feedback.

**Comment 2:** ESG encompasses corporate performance across environmental, social, and governance dimensions, with the social component being most closely associated with CSR. However, the results presented in Table 10 indicate that NAMR demonstrates no significant impact on social performance. The author needs to provide an explanation for this finding.

Response：Thank you for your insightful comment .We have provided the following explanations for this finding:

Since NAMR restricts speculative financial activities, it forces firms to allocate resources more efficiently, influencing their engagement with ESG-related CSR initiatives. The reduction in financial flexibility might lead firms to prioritize governance compliance (G) and environmental (E) initiatives over voluntary social contributions (S). Under an ESG-driven CSR approach, firms must balance financial discipline with sustainability—meaning that governance (G) and environmental (E) commitments become more directly measurable under ESG frameworks. However, social responsibility (S) efforts may remain voluntary, which could explain why NAMR's impact on the S-dimension of CSR is weaker.

Delayed Impact on Social Performance: Unlike environmental initiatives (which require immediate investment) and governance compliance (which is highly regulated), social responsibility investments often have longer adjustment periods. NAMR may trigger changes that manifest beyond the observed time frame，the NAMR may trigger changes that manifest outside the time frame of observation. To further explore this, we conducted an additional experiment where we regressed social performance (S) lagged by one period. The results show a significant negative effect of NAMR on lagged social performance (L.S) (p<0.1). This suggests that the overall non-significance of the social dimension may be due to the lagged policy effect of NAMR, rather than a failure of the theoretical mechanism. This theory suggests that changes may take time to fully appear in the data.

For the clarity and readability of the paper, we included the lagged item regression results in the response to the review rather than in the revised manuscript draft. The results are presented below:

|  | (1) | (2) |
| --- | --- | --- |
| VARIABLES | S | L.S |
|  |  |  |
| Post*Prefin | -.00019 | -0.053*** |
|  | (-0.01) | (-4.18) |
| Size | 0.393*** | 0.297*** |
|  | (12.12) | (8.89) |
| Lev | -0.074*** | -0.091*** |
|  | (-3.83) | (-4.79) |
| ROA | 0.014 | -0.016 |
|  | (1.07) | (-1.31) |
| Cashflow | 0.018* | 0.013 |
|  | (1.87) | (1.53) |
| Growth | -0.790* | -0.079 |
|  | (-1.79) | (-0.19) |
| Board | -0.040** | -0.044*** |
|  | (-2.44) | (-2.84) |
| Indep | 0.013 | 0.011 |
|  | (0.95) | (0.83) |
| Top10 | -0.027 | 0.004 |
|  | (-1.01) | (0.17) |
| Balance | 0.036** | 0.007 |
|  | (2.12) | (0.44) |
| INST | 0.066** | 0.018 |
|  | (2.32) | (0.66) |
| TMTPay | 0.029* | 0.039** |
|  | (1.74) | (2.56) |
| Separate | 0.003* | 0.003 |
|  | (1.69) | (1.50) |
| Constant | -0.317*** | -0.498*** |
|  | (-13.40) | (-26.38) |
| Observations | 6,820 | 5,867 |
| R-squared | 0.374 | 0.443 |
| FIRM FIXED EFFECT | Yes | Yes |
| Year Fixed Effect | Yes | Yes |

t-statistics in parentheses

*** p<0.01, ** p<0.05, * p<0.1
